# Hydrophilic Modification of Polytetrafluoroethylene (PTFE) Capillary Membranes with Chemical Resistance by Constructing Three-Dimensional Hydrophilic Networks

**DOI:** 10.3390/polym16081154

**Published:** 2024-04-19

**Authors:** Mingpeng Hou, Qiuying Li, Yanchao Che

**Affiliations:** 1 School of Material Science and Engineering, East China University of Science and Technology, Shanghai 200237, China; y30210764@mail.ecust.edu.cn; 2 Zhenjiang Fluorine Innovation Material Technology Co., Ltd., Danyang 212322, China

**Keywords:** PTFE capillary membrane, hydrophilic modification, water filtration, chemical resistance

## Abstract

Polytetrafluoroethylene (PTFE) capillary membranes, known for the great chemical resistance and thermal stability, are commonly used in membrane separation technologies. However, the strong hydrophobic property of PTFE limits its application in water filtration. This study introduces a method whereby acrylamide (AM), N, N-methylene bisacrylamide (MBA), and vinyltriethoxysilane (VTES) undergo free radical copolymerization, followed by the hydrolysis-condensation of silane bonds, resulting in the formation of hydrophilic three-dimensional networks physically intertwined with the PTFE capillary membranes. The modified PTFE capillary membranes prepared through this method exhibit excellent hydrophilic properties, whose water contact angles are decreased by 24.3–61.2%, and increasing pure water flux from 0 to 1732.7–2666.0 L/m^2^·h. The enhancement in hydrophilicity of the modified PTFE capillary membranes is attributed to the introduction of hydrophilic groups such as amide bonds and siloxane bonds, along with an increase in surface roughness. Moreover, the modified PTFE capillary membranes exhibit chemical resistance, maintaining the hydrophilicity even after immersion in strong acidic (3 wt% HCl), alkaline (3 wt% NaOH), and oxidative (3 wt% NaClO) solutions for 2 weeks. In conclusion, this promising method yields modified PTFE capillary membranes with great hydrophilicity and chemical resistance, presenting substantial potential for applications in the field of water filtration.

## 1. Introduction

Currently, water shortage caused by industrial waste water discharge and oil spills is a global concern, causing severe environmental pollution. Sustainable water production has emerged as a priority in current research [1,2,3].

The membrane separation technology presents promising applications due to its high efficiency, low energy consumption, low cost-effectiveness, and low secondary pollution [4,5]. In recent decades, membrane separation has become an environmentally friendly, energy-efficient separation and purification technology, which makes it suitable for large-scale waste water treatment [6,7]. Polymeric membranes like polypropylene [8], polyethersulfone [9,10], polyvinylidene difluoride [11,12], and PTFE [13,14,15,16] have gained wide usage. In particular, PTFE is known for its excellent chemical resistance, thermal stability, aging resistance, and electrical insulation properties, and is widely used in the fields of aerospace, automotive, and environmental industries [17,18,19]. PTFE membranes have been successfully used in membrane bioreactor technology for waste water treatment and oil–water separation [20,21,22]. Sun et al. combined carboxymethyl cellulose solution using water as the solvent, with a PTFE membrane to obtain a modified membrane that exhibited excellent oleophobic property [20]. Kim et al. adopted a strategy of using an anaerobic fluidized bed biofilm reactor with a hydrophobic PTFE membrane structure to treat waste water and evaluated the feasibility of post-treatment of anaerobic biological waste. They observed that the permeation flux of PTFE membrane just decreased to 84% of the initial flux [22].

PTFE membranes can be classified into flat membranes and tube-shaped membranes according to their geometric shapes. Tube-shaped membranes can be roughly classified into three categories based on their different diameters: tubular membranes with a diameter larger than 10 mm, capillary membranes with a diameter ranging from 0.5 mm to 10 mm, and hollow fiber membranes with a diameter smaller than 0.5 mm. Different diameters of PTFE tubular membranes are suited for various scales of fluid control and filtration scenarios. Thus, the selection of the appropriate diameter of PTFE tubular membranes in practical applications depends on specific conditions to meet the desired requirements. In the case of PTFE capillary membranes, they excel in precise control and analytical filtration of minute liquid volumes compared to larger diameter tubular membranes. In contrast to PTFE hollow fiber membranes, the pores on PTFE capillary membranes are larger, reducing the chance of membrane blockage during subsequent modifications.

However, the completely symmetric carbon–fluorine bond structure and the extremely low surface energy result in strong hydrophobicity of PTFE capillary membranes, limiting its application in water filtration [23,24]. Thus, hydrophilic modifications of PTFE capillary membranes become necessary. Creating a hydrophilic interface on the capillary membrane surface effectively prevents or reduces the adsorption or deposition of pollutants. Common methods for hydrophilic modification of PTFE capillary membranes include radiation grafting, plasma grafting, wet chemical treatment, surfactant coating, and deposition modification [25,26,27,28,29,30,31,32,33,34,35,36,37]. Yet, these methods have their limitations such as potential harm to human health, equipment demands, and environmental pollution.

Surface crosslinking, a physical modification method, involves intertwining hydrophilic substances around membrane fibers to form a stable structure [38,39,40,41]. Wang intertwined hydrophilic polyvinyl alcohol on PTFE membranes using electrospinning, followed by glutaraldehyde crosslinking, creating a dual-layer membrane resistant to oil contamination [38]. For the membranes, there is still room for further improvement in chemical resistance. Liu developed a hydrophilic crosslinked network using polyvinylpolypyrrolidone with great hydrophilicity and vinyltriethoxysilane acting as a crosslinker, showing the water flux of 200 L/m^2^·h [39]. However, hydrophilic PTFE membranes prepared under this experimental conditions cannot currently achieve both high water flux and great chemical resistance. Hydrophilic groups can intertwine more densely at the fiber nodes of the PTFE capillary membrane. Perhaps using agents with better hydrophilic groups and further forming three-dimensional networks to enhance stability may be an alternative solutions.

This study proposes a straightforward approach by constructing a hydrophilic three-dimensional network physically intertwined with pristine PTFE capillary membranes, resulting in hydrophilic modified PTFE capillary membranes. AM and MBA are used as hydrophilic agents containing amide groups. They undergo free radical copolymerization with VTES, coupled with the hydrolysis and condensation of silane bonds, constructing a hydrophilic three-dimensional network intertwined with pristine PTFE capillary membranes. The modified PTFE capillary membranes exhibit excellent hydrophilic properties, high pure water flux, and great chemical resistance.

## 2. Materials and Methods

### 2.1. Materials

Triethoxyvinylsilane, 97%, and triethyl phosphate, GR, Shanghai Maclin Biochemical Technology Co., Ltd., Shanghai, China; Acrylamide, AR, and alcohol, purity ≥99.8%, Shanghai Aladdin Biochemical Technology Co., Ltd., Shanghai, China; N,N′-Methylene bisacrylamide, 98%, and citric acid monohydrate, 98%, Shanghai Haohong Biomedical Technology Co., Ltd., Shanghai, China; Azobisisobutyronitrile, 99%, Beijing Bailingwei Technology Co., Ltd., Beijing, China; PTFE capillary membrane, outer diameter 2.2 mm, inner diameter 1.1 mm, Danyang Keer Electronic Technology Co., Ltd., Zhenjiang, China; Bovine serum albumin, BS114, Shanghai Jinpan Biotechnology Co., Ltd., Shanghai, China.

### 2.2. Preparation of Hydrophilic PTFE Capillary Membranes

**Scheme 1:**
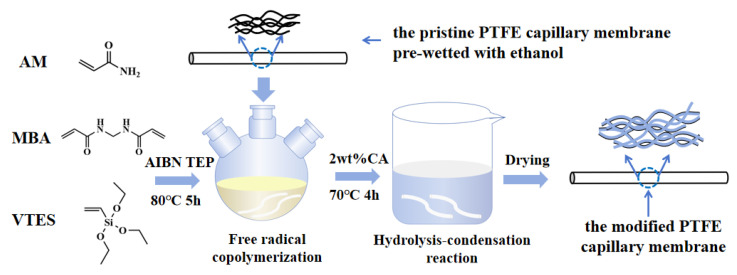
Schematic illustration of the preparation process for the hydrophilic modified PTFE capillary membranes.

The sample preparation process is illustrated in Scheme 1. PTFE capillary membranes were thoroughly washed and then immersed in an infiltrating solution, ensuring complete wetting to exhibit a transparent and colorless state. Amounts of 11.20 g of VTES, 1.50 g of amide hydrophilic agents (AM and MBA), and 0.08 g of azobisisobutyronitrile (AIBN) were added to a three-neck flask with 100 g of triethyl phosphate (TEP). Subsequently, the PTFE capillary membranes pre-wetted with ethanol were placed into the three-neck flask and stirred at room temperature under nitrogen protection for 30 min. Then, the reaction continued for 5 h at 80 °C, yielding a pale white precursor polymer solution. The PTFE capillary membranes were then transferred to a 2.0% mass fraction solution of citric acid monohydrate at 70 °C for 4 h to complete the hydrolysis-condensation reaction of siloxane through hydrothermal treatment. Finally, the PTFE capillary membranes were removed, washed, and dried to obtain the hydrophilic PTFE capillary membranes for subsequent characterization.

Five different samples were prepared, named as PTFE-AM1.50-MBA0, PTFE-AM1.00-MBA0.50, PTFE-AM0.75-MBA0.75, PTFE-AM0.50-MBA1.00, and PTFE-AM0-MBA1.50, which was based on varying hydrophilic agent amounts. Formulations for PTFE capillary membranes are listed in Table 1.

### 2.3. Characterization of Structure and Surface Chemical Composition of PTFE Capillary Membranes

Surface chemical composition of the prepared membranes was measured using X-ray photoelectron spectroscopy (XPS, PerkinElmer PHI 5000C ESCA System with Mg/Al dual anode Hel/Helium UV source, Kratos, XSAM800, Cambridge, MA, USA). The surface chemical structure of the membranes was studied using attenuated total reflection Fourier-transform infrared spectroscopy (ATR-FTIR, Nicolet 5700, Cambridge, MA, USA) over a range of 4000–400 cm^−1^. The surface morphologies of the pristine PTFE capillary membrane and modified membranes were observed using field emission scanning electron microscopy (FESEM, HitachiS-4800, Tokyo, Japan). The membrane was sputtered with gold before morphology observation. The accelerator voltage in SEM observation was set to 4.0 kV.

### 2.4. Surface Wettability and Pure Water Flux Tests of PTFE Capillary Membranes

The surface wettability was characterized by the water contact angle on the membrane surface. The water contact angle on the membrane surface was measured using a static water contact angle measuring instrument (WCA, JY82B, China). Before the test, the membrane was cut along the diameter direction, and then the membrane was placed flat on the platform. The outer surface of the membranes faced up, and the two ends were fixed. For each sample, five membrane measurements were taken and then averaged, and the standard deviations were calculated. Deionized water (3 μL) was dropped onto the membrane surface at room temperature. The average of five measurements was taken.

**Scheme 2:**
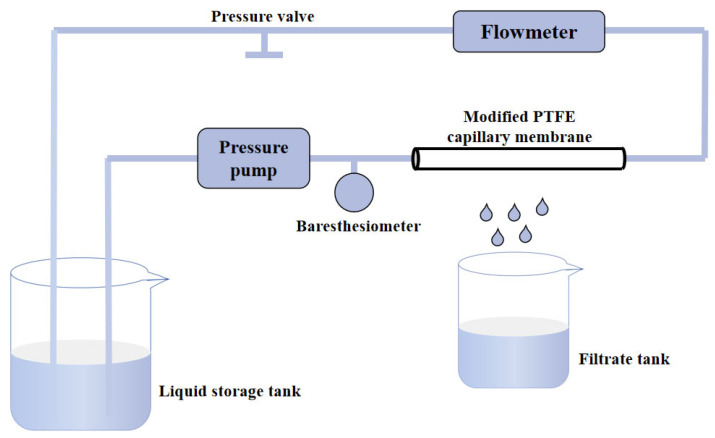
Schematic diagram of filtering device.

Using a flow filtration system (moist-02hfm, China) shown in Scheme 2, the pure water flux of the PTFE capillary membrane was tested. Before the test, the membrane specimen loaded in the filtration pool was pressurized with deionized water at 0.2 MPa for at least an hour to ensure stable membrane flux. Pure water flux (*J*_w_) was measured using deionized water under a transmembrane pressure of 0.1 MPa, which was calculated using the Formula (1)

(1) $$J_{w}=\frac{V}{A\times \Delta t}$$

where *J*_w_ is the volume permeate pure water flux (L/m^2^·h), *V* is the permeate volume (L) within time interval Δt (h), and *A* is the effective permeation area of the membrane (m^2^).

### 2.5. Filtration Performance Tests of Hydrophilic Modified PTFE Capillary Membranes

BSA solution was used to test the filtration performance of the modified membranes. An amount of 1.0 g/L BSA solution was prepared, and filtration experiments were performed under a transmembrane pressure of 0.1 MPa using the device shown in Scheme 2. The solutions before and after filtration were analyzed using total organic carbon (TOC, TOC-3000, Shanghai, China) analyzer. The filtration time of each sample was 24 h. An amount of 10 mL of liquid was collected from the storage tank and filtrate tank, respectively, under the condition of stable flow rate for testing after 24 h. The average of three measurements was taken. Equation (2) was used to calculate the rejection of membranes.

(2) $$R=(1-\frac{C_{1}}{C_{0}})\times 100\%$$

where *R* is the rejection, *C*_1_ and *C*_0_ are the carbon concentration after and before filtration.

### 2.6. Chemical Resistance Tests of Hydrophilic Modified PTFE Capillary Membranes

Hydrophilic PTFE capillary membranes were subjected to acid, alkali, and oxidation resistance tests. Acid resistance testing involved immersing the modified PTFE capillary membrane in the 3% mass fraction HCl solution, alkali resistance testing involved immersion in the 3% mass fraction NaOH solution, oxidation resistance testing involved immersion in the 3% mass fraction NaClO solution for two weeks, and then testing water contact angles and pure water flux once a week.

## 3. Results and Discussion

### 3.1. Mechanism for Intertwining the Hydrophilic Three-Dimensional Network with PTFE Capillary Membranes

**Figure 1:**
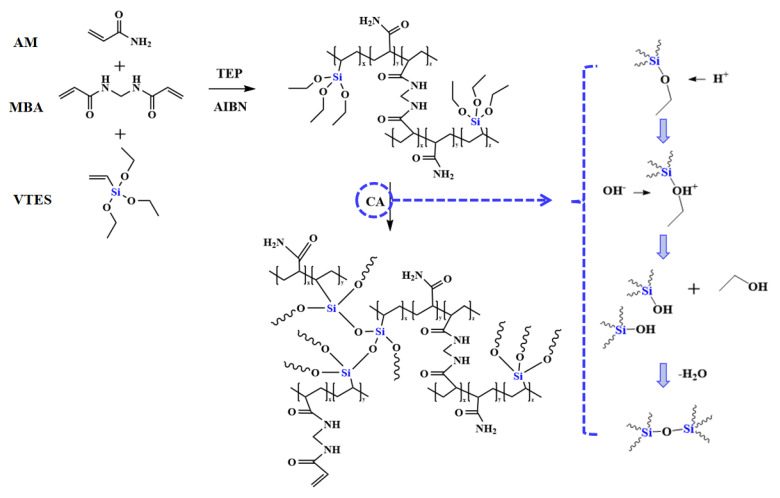
Chemical reaction formula of the synthesis of the hydrophilic three-dimensional network.

The construction of a hydrophilic three-dimensional network shown in Figure 1. involves free radical copolymerization and hydrolytic condensation of silane bonds. AM, MBA, and VTES undergo copolymerization. Both AM and MBA contain amide groups with conjugated double bonds, exhibiting electron conjugation and electron-withdrawing inductive effects. Homolysis of double bonds occurs during polymerization, forming free radicals. VTES can also undergo free radical polymerization due to its structural asymmetry. The copolymerization introduces hydrophilic amide groups and the long-chain polymers interpenetrate PTFE capillary membranes. Multiple-chain molecules form a three-dimensional network structure on the membranes, increasing the density of hydrophilic groups at fiber nodes.

When the PTFE capillary membranes were immersed in a 2 wt% CA solution, the H^+^ in the solution binds to the negatively charged oxygen atom in the siloxane, forming an intermediate Si-OH-CH_2_-CH_3_ structure. When -CH_2_-CH_3_ and -OH combine to separate CH_3_CH_2_OH molecules, the OH^−^ in the solution combines with positively charged Si atoms to form silanols (Si-OH). Subsequently, under acidic conditions, silanols condense with each other, resulting in producing Si-O-Si structures, which can firmly anchor hydrophilic groups onto the PTFE capillary membranes.

### 3.2. Chemical Composition of Modified PTFE Capillary Membranes

**Figure 2:**
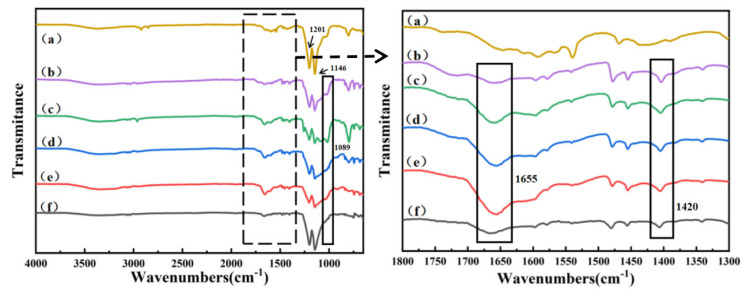
FTIR spectra of (**a**) the pristine PTFE capillary membrane, (**b**) PTFE-AM1.50-MBA0, (**c**) PTFE-AM1.00-MBA0.50, (**d**) PTFE-AM0.75-MBA0.75, (**e**) PTFE-AM0.50-MBA1.00, and (**f**) PTFE-AM0-MBA1.50.

To validate the formation of a hydrophilic three-dimensional network physically intertwined on the surface of the PTFE capillary membrane, ATR-FTIR characterization tests were conducted on the samples, as shown in Figure 2.

From Figure 2a, it can be seen that the pristine PTFE capillary membrane displayed sharp peaks at 1146 cm^−1^ and 1201 cm^−1^, corresponding to the stretching vibration of -CF_2_ [42]. Observing Figure 2b–f, it was found that the stretching vibration peak of -CF_2_ weakened, and a new characteristic peak at 1089 cm^−1^ emerged, attributed to the Si-O-Si bond formed after the hydrolytic condensation of VTES [43]. The adsorption peak at 1655 cm^−1^ corresponds to the stretching vibration of the C=O bond in the amide group, while the peak at 1420 cm^−1^ corresponds to the stretching vibration of the C-N bond in the amide group, which corresponded well with the existing literature [42,43]. The results indicated the successful introduction of the amide groups onto the PTFE capillary membranes.

The modified membranes exhibited a more pronounced stretching vibration peak of the C=O bond in the amide group as the MBA content in the formulation increased. This enhancement arises because both ends of the MBA molecule can participate in free radical polymerization, and connect two linear polymer chains through bonding, which thereby can generate a network structure. Consequently, more hydrophilic groups physically intertwine on the surface of the PTFE capillary membranes. However, for the PTFE-AM0-MBA1.50 sample containing only MBA as a hydrophilic agent, the stretching vibration peak weakened. MBA’s special structure with double bonds at both ends allows it to generate an overly dense three-dimensional cross-linked network during free radical polymerization. The amide group is encapsulated by a large number of other groups, which suggests a reduction in the exposed amide groups on the PTFE capillary membranes, which can affect the hydrophilic effect [39].

**Figure 3:**
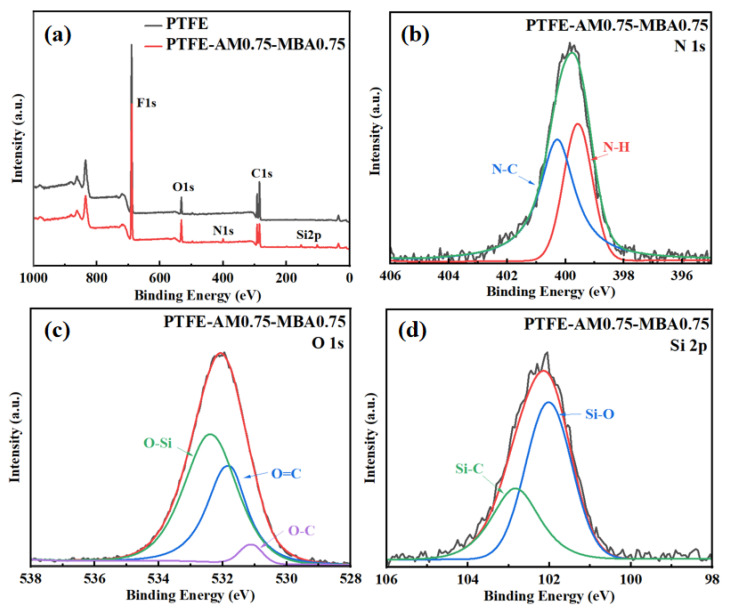
Surface chemistry characterizations of (**a**) wide-scan spectra, (**b**) N 1s, (**c**) O 1s, and (**d**) Si 2p core-level signals of XPS spectra for PTFE and PTFE-AM0.75-MBA0.75.

For further validation of the modified PTFE capillary membrane’s surface chemical structure, XPS characterization tests were performed on the pristine PTFE capillary membrane and PTFE-AM0.75-MBA0.75. The wide-scan spectrum is shown in Figure 3a. For the pristine PTFE capillary membrane, absorption peaks appeared at 290 eV and 695 eV, corresponding to C 1s and F 1s, respectively. PTFE-AM0.75-MBA0.75 exhibited two new element peaks at 102 eV and 400 eV, corresponding to Si 2p and N 1s [42]. The Si element comes from VTES involved in free radical polymerization, while the N element comes from AM and MBA. This indicates the existence of VTES, AM, and MBA onto the PTFE capillary membrane. Additionally, Table 2 lists the elemental composition of the pristine PTFE capillary membrane and PTFE-AM0.75-MBA0.75, consistent with the results shown in the XPS wide-scan spectrum.

XPS spectra for PTFE capillary membranes were shown in Figure 3. Peak fitting was performed for N 1s, O 1s, and Si 2p of PTFE-AM0.75-MBA0.75, as depicted in Figure 3b–d. From Figure 3b, it can be observed that the core-level spectrum of N 1s exhibited peaks at 399.7 eV and 400.4 eV, corresponding to N-H and N-C, respectively, originating from the amide bonds in AM and MBA, which are concurrent with the existing literature [42]. In Figure 3c, O 1s displayed peaks at 530.2 eV, 531.8 eV, and 532.9 eV. The peak at 532.9 eV corresponds to the Si-O-Si structure formed after the hydrolytic condensation of VTES, the peak at 531.8 eV is attributed to the C=O in the amide bonds, and the peak at 530.2 eV for the C-O structure comes from the unhydrolyzed ethoxy groups of VTES. The data and the values found in the literature are concordant [39]. In Figure 3d, Si 2p exhibited peaks at 102.0 eV and 102.8 eV, corresponding to Si-O and Si-C from VTES [44]. The peak fitting results confirm the successful introduction of amide and siloxane bonds onto the PTFE capillary membranes, consistent with the front ATR-FTIR characterization tests.

### 3.3. Surface Morphology of Modified PTFE Capillary Membranes

**Figure 4:**
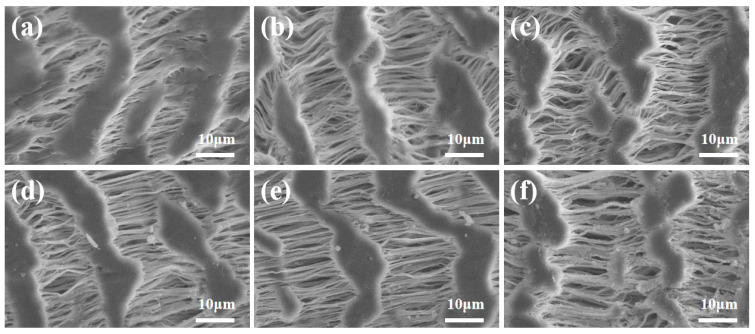
SEM images of (**a**) pristine PTFE capillary membrane, (**b**) PTFE-AM1.50-MBA0, (**c**) PTFE-AM1.00-MBA0.50, (**d**) PTFE-AM0.75-MBA0.75, (**e**) PTFE-AM0.50-MBA1.00, and (**f**) PTFE-AM0-MBA1.50.

Figure 4 depicts SEM images of the PTFE capillary membranes before and after hydrophilic modification. Each sample group exhibited regularly arranged porous structures, which comprise numerous PTFE fibers and nodes. Notably, there were no significant structural changes in the PTFE capillary membrane before and after modification. This suggests that the hydrophilic modification method does not change the pristine porous structure of the PTFE capillary membranes. The surface of the pristine PTFE capillary membrane (as seen in Figure 4a) appeared relatively smooth. However, an observation of Figure 4b–f revealed that the surface became rough. SEM images of the membrane were taken to assess the depth of modification [42]. From the SEM images, it could be observed that the fibers of the modified PTFE capillary membrane became thicker and irregularly shaped particles were attached to it, forming a hydrophilic network physically entangled at fiber nodes. This could reflect the generation of hydrophilic three-dimensional networks. After the free radical copolymerization and silane hydrolysis, a hydrophilic three-dimensional network adheres and intertwines around the PTFE fiber, resulting in granular aggregation on the PTFE fibers. Surface morphology observed by SEM provides initial evidence for supporting the successful preparation of modified PTFE capillary membranes using AM, MBA, and VTES as hydrophilic agents. With an increase in MBA content in the formulation, the aggregation of granules on the PTFE fiber surface increased. This is due to the presence of two double bonds in each MBA molecule, enabling the formation of a three-dimensional molecular network by linking two linear polymer chains through free radical copolymerization. As the MBA content in the formulation increases, more linear polymer chains connect to form a denser three-dimensional network [27], leading to an uneven agglomeration. The increase in crosslinking sites and granular aggregation on PTFE fibers may lead to pore blockage, and excessive granular aggregation could potentially affect the hydrophilicity and water flux of the PTFE capillary membranes.

### 3.4. Surface Wettability and Filtration Performance Tests of PTFE Capillary Membranes

**Figure 5:**
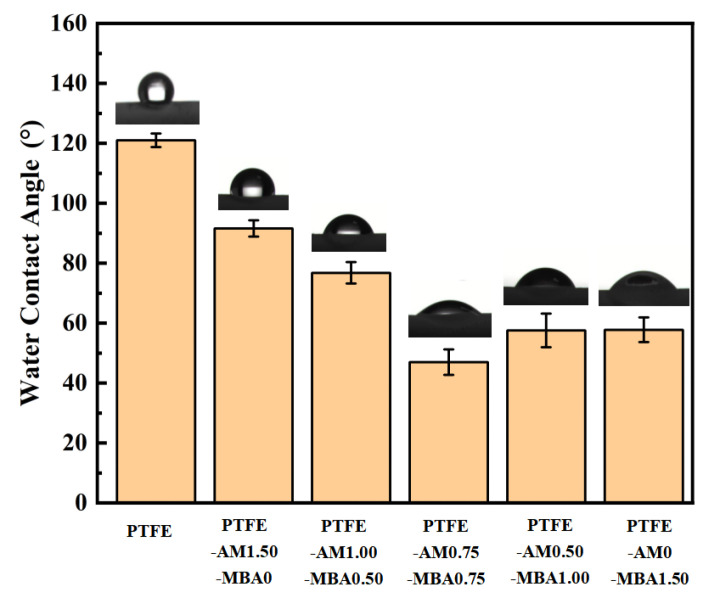
Instantaneous water contact angles of PTFE capillary membranes before and after hydrophilic modification.

The instantaneous water contact angles of the PTFE capillary membranes before and after hydrophilic modification are depicted in Figure 5, with detailed data provided in Table 3. The pristine PTFE capillary membrane exhibited a high water contact angle of 120°, demonstrating strong hydrophobicity. As the MBA content in the formulation increased, the water contact angle of the hydrophilic PTFE capillary membrane declined. Notably, PTFE-AM0.75-MBA0.75 exhibited the lowest water contact angle of 42°, displaying excellent hydrophilicity compared to the pristine membrane. The greater the level of introduction of amide bond groups onto the membrane surface increases its hydrophilicity, leading to enhanced hydrophilic effects with higher MBA concentrations. This improvement originated from the higher concentration of MBA which facilitate the formation of a molecular network through free radical copolymerization, allowing more hydrophilic groups to intertwine closely on the PTFE capillary membrane. The increased number of hydrophilic groups contributed to the reduction in water contact angle. Simultaneously, granular aggregation on the surface of the PTFE capillary membrane can increase the surface roughness, enhancing hydrophilicity of the membranes.

Both AM and MBA contain amide groups which can be introduced into the long-chain molecules generated through free radical polymerization and physically intertwined onto the fiber nodes of the PTFE capillary membrane. The amide groups, as polar groups, improved the wetting properties of the PTFE capillary membrane, enhancing its hydrophilicity. The combined action of free radical polymerization and hydrolytic condensation resulted in the creation of a hydrophilic three-dimensional network, physically intertwined onto the fiber nodes of the PTFE capillary membrane, roughening the originally smooth PTFE surface. An increased surface roughness led to an increased surface area per unit volume. This increase in roughness implies unevenness on the membrane surface, exposing more polar groups to water molecules, thereby enhancing the hydrophilic properties of the PTFE capillary membrane [29,30].

However, with a further increase in MBA content in the formulation, the water contact angle increased instead, rising to 60° for the PTFE-AM0-MBA1.50 sample. Despite this, compared to the pristine PTFE capillary membrane, the PTFE-AM0-MBA1.50 sample still exhibited good hydrophilicity. It is because a higher MBA content led to increased cross-linking and granular aggregation on PTFE fibers, resulting in pore blockage. Meanwhile, an infrared analysis indicated a decrease in the exposed amide groups on the surface of the PTFE capillary membrane, reducing the hydrophilic groups and thus reducing the hydrophilic effect.

**Figure 6:**
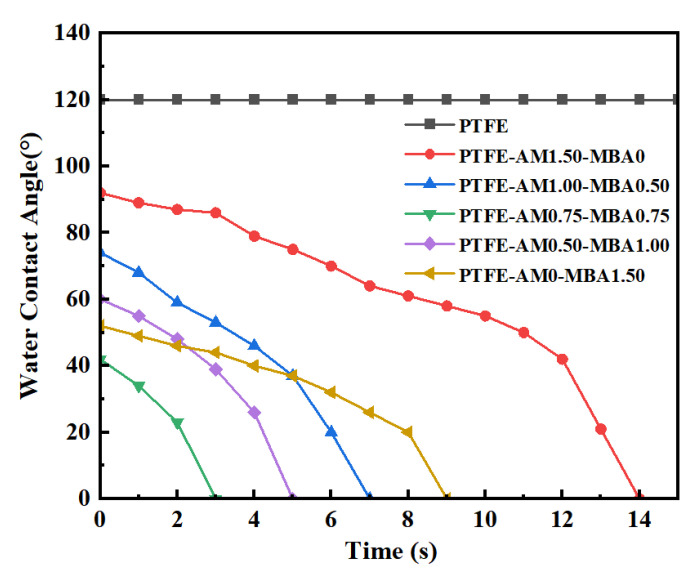
Time-dependent curves of water contact angles of PTFE capillary membranes before and after hydrophilic modification.

Figure 6 illustrates the change in water contact angle over time for the PTFE capillary membranes before and after hydrophilic modification. The pristine PTFE capillary membrane’s water contact angle remained stable at 120°, indicating consistent hydrophobicity. The water contact angle of the PTFE capillary membrane gradually decreased over time after modifications, eventually allowing complete water infiltration. Among all modified membranes, PTFE-AM0.75-MBA0.75 exhibited the smallest instantaneous water contact angle and the shortest infiltration time, with a contact angle of 42°, achieving complete infiltration within 3 s. This was due to the more intensive hydrophilic three-dimensional network formed as the MBA content in the formulation increased, allowing more hydrophilic groups to physically intertwine on the PTFE capillary membrane and providing the modified membrane with outstanding hydrophilicity. Conversely, for the PTFE-AM0-MBA1.50 sample, despite its notable hydrophilicity, the complete infiltration time was relatively longer, taking 14 s, which was attributed to the pore blockage.

**Figure 7:**
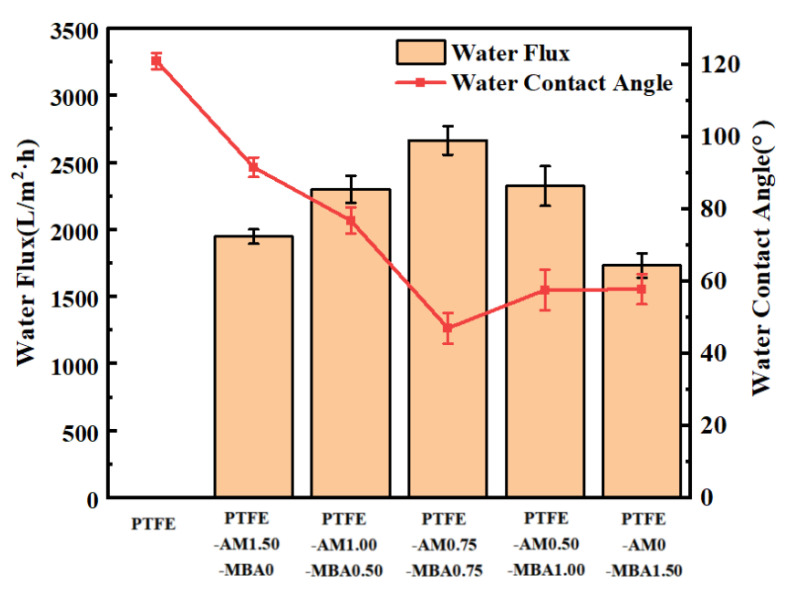
Water flux and water contact angles of the pristine PTFE capillary membrane and hydrophilic modified PTFE capillary membranes.

Figure 7 presents the water flux and water contact angle test outcomes for the pristine PTFE capillary membrane and the hydrophilic modified PTFE capillary membranes. Specific numerical values for water flux and water contact angle are detailed in Table 3. The pristine PTFE capillary membrane demonstrated strong hydrophobicity, resulting in a measured water flux of 0 during testing. The water flux of PTFE-AM1.50-MBA0 reached 1949.7 L/m^2^·h. Subsequently, PTFE-AM1.00-MBA0.50 exhibited increased water flux at 2301.5 L/m^2^·h. The highest water flux observed was with PTFE-AM0.75-MBA0.75 at 2666.0 L/m^2^·h. The investigation revealed a progressive increase in the water flux of modified PTFE capillary membranes as the MBA content in the formulation increased, consistent with the results from the water contact angle tests. However, with a further increase in MBA content, a decrease in water flux was observed. Specifically, the water flux of PTFE-AM0.50-MBA1.00 and PTFE-AM0-MBA1.50 was 2325.1 L/m^2^·h and 1732.7 L/m^2^·h, respectively.

Overall, the water flux of the modified PTFE membranes initially increased and then decreased with the increasing MBA content in the formulation. The increasing trend can be attributed to the higher MBA concentrations favoring the attachment of more hydrophilic groups on the membrane’s fibers and nodes, which can enhance the surface hydrophilicity and increase the pure water flux [39]. However, a further rise in MBA content led to excessive participation in free radical copolymerization, resulting in the formation of an overly dense hydrophilic three-dimensional network. This network excessively aggregated particles on the surface of the PTFE capillary membrane, causing the pore blockage and subsequently reducing the water flux of the modified PTFE capillary membrane despite increased surface roughness.

**Figure 8:**
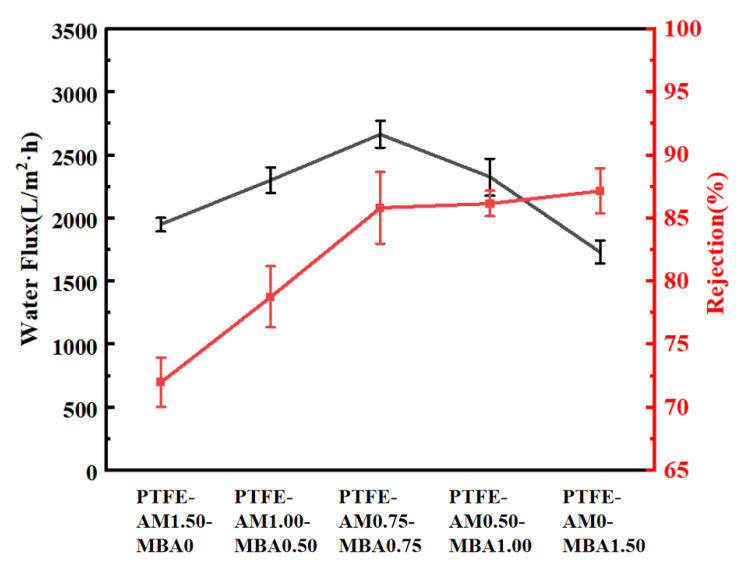
Solution flux and rejection of BSA solution of hydrophilic modified PTFE capillary membranes.

Figure 8 presents the water flux and rejection of the BSA solution of hydrophilic modified PTFE capillary membranes. The oil content in the filtrate was detected using TOC. The difference in flux when filtering BSA aqueous solution and pure water filtration was very small, in which PTFE-AM0.75-MBA0.75 achieved the maximum flux of 2603.5 L/m^2^·h. The solution flux of the modified membrane did not significantly decrease within 24 h, demonstrating good stability performance, which was attributed to excellent hydrophilicity. The rejection rate of the modified PTFE capillary membrane increased gradually with the increase in MBA content in the formulation. The maximum rejection was up to 87.2%, which shows good filtration performance.

### 3.5. Chemical Resistance of Hydrophilic Modified PTFE Capillary Membranes

**Figure 9:**
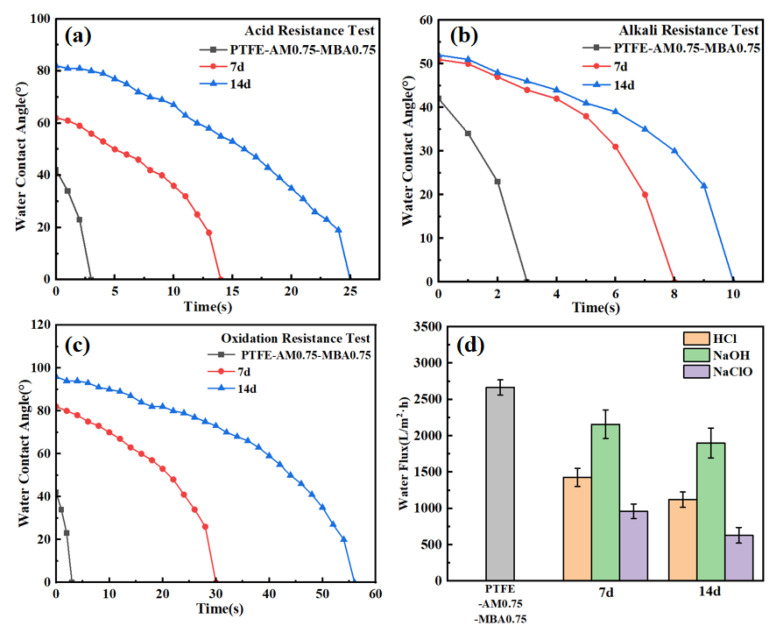
Time-dependent curves of water contact angles of PTFE-AM0.75-MBA0.75, which were treated by immersing in a 3% mass fraction (**a**) HCl solution, (**b**) NaOH solution, and (**c**) NaClO solution for 2 weeks and (**d**) the change in water flux.

In practical filtration applications, PTFE capillary membranes often encounter harsh chemical environment involving strong acids, bases, and oxidants. Therefore, the modified PTFE capillary membranes are required to exhibit resistance to these environments. This study investigated the chemical resistance of the modified PTFE capillary membranes. PTFE-AM0.75-MBA0.75, showing great hydrophilicity, was immersed in solutions of HCl (3 wt%), NaOH (3 wt%), and NaClO (3 wt%) for two weeks, followed by measurements of the water contact angle and water flux of the membranes, whose data are shown in Table 4 and Figure 9.

Overall, PTFE-AM0.75-MBA0.75 retained good hydrophilic properties in acidic, alkaline, and oxidizing environments, still allowing water to wet the surface of modified PTFE capillary membranes. After seven days of immersion in HCl, NaOH, and NaClO solutions, the instantaneous water contact angle of PTFE-AM0.75-MBA0.75 slightly increased to 61°, 50°, and 81°, respectively. After 14 days of immersion in above solutions, the instantaneous water contact angle further increased to 82°, 52°, and 96°, with complete wetting achieved within one minute in all cases. After two weeks of immersion in various solutions, the instantaneous water contact angle remained far lower than the water contact angle of the pristine PTFE capillary membrane which was 120°. Meanwhile, PTFE-AM0.75-MBA0.75 maintained high water flux after chemical resistance testing. Specifically, after seven days of immersion in HCl, NaOH, and NaClO solutions, the pure water flux for PTFE-AM0.75-MBA0.75 was 1429.1 L/m^2^·h, 2157.9 L/m^2^·h, and 959.7 L/m^2^·h, respectively. After 14 days of immersion in HCl, NaOH, and NaClO solutions, the flux reduced to 1121.6 L/m^2^·h, 1900.8 L/m^2^·h, and 631.3 L/m^2^·h. Although the decrease in pure water flux was relatively high, PTFE capillary membranes maintained high flux compared to the pristine PTFE capillary membrane with a flux of 0 L/m^2^·h.

**Figure 10:**
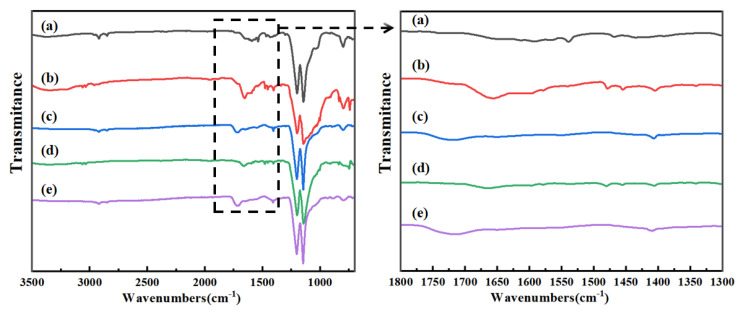
FTIR spectra of (**a**) the pristine PTFE capillary membrane, (**b**) PTFE-AM0.75-MBA0.75, and PTFE-AM0.75-MBA0.75 which were treated by immersing in a 3% mass fraction (**c**) HCl solution, (**d**) NaOH solution, and (**e**) NaClO solution for 2 weeks.

FTIR spectra of the pristine PTFE capillary membrane and PTFE-AM0.75-MBA0.75 before and after chemical resistance tests are shown in Figure 10. From Figure 10c–e, it can be seen that the peak of the Si-O-Si bond at 1089 cm^−1^, and C-N bond at 1420 cm^−1^ still existed. The hydrophilic membranes prepared by this modification method were able to remain stable under harsh chemical conditions. The three-dimensional network remained stable under strong acid, alkali, and oxidative environments, which gave the PTFE capillary membranes great chemical resistance.

It suggested that the physically intertwined hydrophilic three-dimensional network with the PTFE capillary membrane was not entirely removed. Even in harsh chemical environments, this network remained firmly anchored to the membranes. The reason why modified PTFE capillary membranes have chemical resistance is that the three-dimensional hydrophilic network generated by free radical copolymerization contains amide groups and silicon–oxygen bonds, which themselves have chemical resistance. Additionally, the physical intertwinement of the three-dimensional hydrophilic network reduces contact between strong acids, bases, and oxidizing agents with more hydrophilic groups, thus shielding inner hydrophilic groups from aggressive chemical exposure. Essentially, this network restricts the diffusion of small chemical molecules within the PTFE capillary membrane, preventing the degradation of the physically intertwined polymer hydrophilic network.

**Figure 11:**
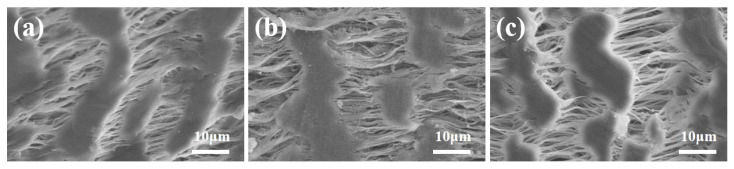
SEM images of PTFE-AM0.75-MBA0.75, which was treated by immersing in a 3% mass fraction (**a**) HCl solution, (**b**) NaOH solution, and (**c**) NaClO solution for 2 weeks.

Compared to acid and oxidative resistance, PTFE-AM0.75-MBA0.75 exhibited better alkali resistance. As depicted in Table 4, after 14 days of immersion in a 3 wt% NaOH solution, the instantaneous water contact angle for PTFE-AM0.75-MBA0.75 was only 52°, achieving complete water infiltration within 10 s into the capillary membrane. The flux loss of the modified PTFE capillary membrane was only 28.7%. In contrast, after 14 days of immersion in a 3 wt% HCl solution, the instantaneous water contact angle was 82°, requiring 25 s for complete wetting. The flux loss was 57.9%. After immersion in a 3 wt% NaClO solution for 14 days, the instantaneous water contact angle increased to 96°, requiring 56 s for complete wetting. The flux loss was 76.3%. Figure 11 displays SEM images of PTFE-AM0.75-MBA0.75 after the chemical resistance test for 14 days.

Figure 11a,c revealed rougher and more uneven structures with the appearance of numerous particles reunited. However, in Figure 11b, the morphology of the PTFE capillary membrane which was immersed in the NaOH solution had the close resemblance to the untreated PTFE-AM0.75-MBA0.75, showing minimal surface particles and presenting a smooth and loose porous structure.

For Figure 11a,c, the enhanced alkali resistance of the modified PTFE capillary membrane stems from the greater susceptibility of the carbonyl groups in the amide bonds to acidic conditions and the high oxidative nature of oxidizing agents which can directly degrade bond structures via oxidation reactions. Under alkaline conditions, the oxygen atoms in the amide bonds of AM and MBA acquire negative charges, reducing the nucleophilic attack of hydroxide ions on these bonds, thus lessening the impact of alkaline substances on the amide bonds. Conversely, acidic conditions with hydrogen ions favorably protonate the nitrogen atoms in the amide bonds, causing positive charges on the amide bonds. This protonation weakens the covalent bond between the nitrogen atom and the carbonyl carbon in the amide bonds, leading to increased instability and easier breakage. Additionally, hydrolysis reactions easily occur under acidic conditions, where water molecules react with the carbonyl carbon and nitrogen atoms in the amide bonds, disrupting their structures. Both processes potentially affect the stability of the amide bonds under acidic conditions [34,35,36]. For NaClO solution, which is strongly oxidizing, both amide bonds and siloxane bonds may become unstable under the influence of oxidizing substances. These substances can react with the atoms or structures within these bonds, causing breakage or oxidation, leading to the detachment of the hydrophilic network and affecting its hydrophilic properties. Despite this, the modified PTFE capillary membranes maintain higher water flux and smaller water contact angles compared to the pristine PTFE capillary membrane, demonstrating an acceptable resistance to harsh environments, particularly alkali resistance.

## 4. Conclusions

In conclusion, this study successfully developed modified PTFE capillary membranes with excellent hydrophilic property and chemical resistance. Through the free radical copolymerization of AM, MBA, and VTES as raw materials, coupled with the hydrolysis-condensation reaction of silane bonds, a hydrophilic three-dimensional network was constructed, which could physically entangle with the PTFE capillary membranes, resulting in modified capillary membranes with improved hydrophilic properties. These modified capillary membranes exhibited great hydrophilicity, achieving the best instantaneous water contact angle of 47°, which was decreased by 61.2%, and allowing complete surface wetting within 3 s. Compared to the pristine PTFE capillary membrane, the hydrophilic modification led to higher pure water flux, reaching 2666.0 L/m^2^·h. The introduction of hydrophilic groups such as amide bonds and siloxane bonds, along with increased surface roughness, contributed to the enhanced hydrophilicity of the modified PTFE capillary membranes. Experimental data indicated that PTFE-AM0.75-MBA0.75 exhibited the most effective hydrophilic modification.

After immersion in 3 wt% HCl, NaOH, and NaClO solutions for 14 days, instantaneous water contact angles of PTFE-AM0.75-MBA0.75 were slightly increased to 82°, 52°, and 96°, respectively. At the same time, it was found that the water flux was higher than 631.3 L/m^2^·h, which showed that the modified PTFE capillary membranes still had acceptable hydrophilicity. Further research could focus on the precise control of cross-linked networks and explore the chemical resistance of PTFE capillary membranes in more complex environments to improve their practical feasibility in real-world applications.

## Figures and Tables

**Table 1:** Formulations for different modified PTFE capillary membranes.

	VTES (g)	AM (g)	MBA (g)
PTFE-AM1.50-MBA0	11.20	1.50	0
PTFE-AM1.00-MBA0.50	11.20	1.00	0.50
PTFE-AM0.75-MBA0.75	11.20	0.75	0.75
PTFE-AM0.50-MBA1.00	11.20	0.50	1.00
PTFE-AM0-MBA1.50	11.20	0	1.50

**Table 2:** Elemental composition of the pristine PTFE and PTFE-AM0.75-MBA0.75.

	Content (%)				
	C	N	O	F	Si
Pristine PTFE	22.35	0	6.77	70.88	0
PTFE-AM0.75-MBA0.75	44.14	2.21	9.31	41.64	2.66

**Table 3:** Water flux, water contact angles, and rejection of the pristine PTFE capillary membrane and hydrophilic modified PTFE capillary membranes.

	Water Flux(L/m^2^·h)	Water Contact Angle (°)	Water Contact Angle Decrease Ratio
PTFE	0	121.0 ± 2.2	-
PTFE-AM1.50-MBA0	1949.7 ± 54.3	91.6 ± 2.7	24.3%
PTFE-AM1.00-MBA0.50	2301.5 ± 102.1	76.8 ± 3.6	36.5%
PTFE-AM0.75-MBA0.75	2666.0 ± 105.6	47.0 ± 4.2	61.2%
PTFE-AM0.50-MBA1.00	2325.1 ± 148.1	57.6 ± 5.6	52.4%
PTFE-AM0-MBA1.50	1732.7 ± 91.1	57.8 ± 4.1	52.2%

**Table 4:** Water flux and water contact angles of PTFE-AM0.75-MBA0.75 before and after the chemical resistance test.

Samples		Water Contact Angle (°)	Water Flux (L/m^2^·h)	Flux Loss
PTFE-AM0.75-MBA0.75		47	2666.0 ± 105.6	
7 d	3 wt%HCl	61	1429.1 ± 124.5	45.4%
	3 wt%NaOH	50	2157.9 ± 196.8	19.1%
	3 wt%NaClO	81	959.7 ± 100.4	64.0%
14 d	3 wt%HCl	82	1121.6 ± 106.6	57.9%
	3 wt%NaOH	52	1900.8 ± 205.4	28.7%
	3 wt%NaClO	96	631.3 ± 108.5	76.3%

## Data Availability

Data are contained within the article.
